# Exploring brain changes of impulse control disorders in Parkinson's disease: An ALE study

**DOI:** 10.3389/fnagi.2022.966525

**Published:** 2022-08-30

**Authors:** Lihua Gu, Hao Shu, Yanjuan Wang, Hui Xu

**Affiliations:** ^1^Department of Neurology, Affiliated ZhongDa Hospital, School of Medicine, Southeast University, Nanjing, China; ^2^Department of Neurology, Tianjin Huanhu Hospital, Tianjin, China

**Keywords:** activation likelihood estimation, impulse control disorder, neuroimaging, Parkinson's disease, functional MRI

## Abstract

**Background:**

Previous neuroimaging studies reported inconsistent results for comparison between Parkinson's disease (PD) with impulse control disorder (PD-ICD) and without ICD (PD-no ICD).

**Methods:**

A search was performed in databases (PubMed and Web of Science) to identify studies published before May 2022. An anatomic likelihood estimation (ALE) method study was made for neuroimaging studies in PD-ICD.

**Results:**

The study included 20 studies (including 341 PD-ICD and 437 PD-no ICD). PD-ICD patients showed significant cortical thinning in the right inferior frontal gyrus (IFG), the right middle frontal gyrus (MFG), the left superior frontal gyrus (SFG), the right precentral gyrus (PCG) and the left cingulate gyrus (CG), compared to PD-no ICD patients. The ALE study showed reduced resting-state brain activation in the right IFG, the right PCG, the left insula and the right transverse temporal gyrus (TTG) in PD-ICD, compared to PD-no ICD patients. In addition, PD-ICD showed increased resting-state brain activation in the right caudate, the bilateral insula and the left orbital gyrus (OG), compared to PD-no ICD patients. The study indicated reduced task-related brain activation in the right caudate, the right MFG, the right lentiform nucleus (LN) and the right precuneus (PCUN) in PD-ICD, compared to PD-no ICD patients. The study showed increased task-related brain activation in the left inferior parietal lobule (IPL), the right medial frontal gyrus, the right caudate and the right PCG in PD-ICD, compared to PD-no ICD patients.

**Conclusions:**

The present ALE analysis has confirmed that brain changes in frontal, temporal and basal ganglia regions are among the most frequently reported regions in PD-ICD. Deficits in these regions could play a role in diagnosis of PD-ICD.

## Introduction

Parkinson's disease (PD) is the second most common neurodegenerative disorder that involves the progressive deficit of voluntary motor control (de Lau and Breteler, 2006; Kalia and Lang, 2015). Impulse control disorder (ICD) is an adverse effect of dopaminergic therapy in PD patients. Although ICD is considered as a common non-motor complication of PD, frequency estimates range from approximately 14–60% in PD (Molde et al., 2018). In recent years, ICDs gradually draw attention due to their enormous influence on PD patients and their family (Moegle et al., 2020). ICD patients have trouble in regulating behaviors (Molde et al., 2018), referring to reduced control over compulsive or repetitive behavior and pleasurable feeling when conducting the behavior (Ceravolo et al., 2009). ICDs include pathological gambling (PG), hypersexuality (HS), compulsive eating (CE), compulsive buying (CB).

In recent years, neuroimaging studies [including structural magnetic resonance imaging (MRI), functional MRI (fMRI), positron emission tomography (PET) and single photon emission computed tomography (SPECT)] explored the brain changes of ICD in PD. However, previous neuroimaging studies reported inconsistent results for comparison between PD with ICD (PD-ICD) and without ICD (PD-no ICD). Regarding structural MRI, Biundo et al. (2015) reported significant cortical thinning in fronto-striatal circuitry, especially in the right superior orbitofrontal, left rostral middle frontal, bilateral caudal middle frontal region, and corpus callosum in patients with ICDs, compared to PD-no ICD, whereas Markovic et al. (2017) reported that PD-ICD patients showed cortical thinning of the right pars orbitalis of the inferior frontal gyrus (IFG). Regarding resting state fMRI (rs-fMRI), Tessitore et al. (2017a) reported increased connectivity in the left orbitofrontal cortex and decreased connectivity in the left supramarginal gyrus, the left precuneus (PCUN) in PD-ICD, compared to PD-no ICD patients, whereas Imperiale et al. (2018) found that PD no-ICD patients showed increased functional connectivity of bilateral precentral and postcentral gyri, compared with controls and PD-ICD. These inconsistencies made meta-analysis methods, such as anatomical likelihood estimation (ALE), became an attractive method to recognize trends and convergence across numerous studies. ALE is a coordinate-based meta-analysis method which computes statistically significant foci extracted from different studies to create probability distribution maps for voxels of interest across foci. The probability distribution maps for voxels of interest across foci are then used to generate structural or functional maps across groups of datasets. Thus, the study aimed to conduct a comprehensive review of MRI studies exploring brain changes of ICD in PD.

## Methods

### Search strategy

The present study was conducted according to the Preferred Reporting Items for Systematic Reviews and Meta-Analyses (PRISMA) statement (Moher et al., 2009). Supplementary table S1 illustrated PRISMA checklist. A search was performed in databases (PubMed and Web of Science) to identify studies published before May 2022. Search terms were showed as follows: (“Parkinson's disease” OR “PD”) AND (“impulsive control disorder” OR “pathological gambling” OR “hypersexuality” OR “compulsive eating” OR “compulsive shopping” OR “compulsive buying” OR “punding” OR “compulsive sexual behavior”) AND (“magnetic resonance imaging” OR “neuroimaging” OR “MRI” OR “resting state functional MRI” OR “rs-fMRI” OR “positron emission tomography” OR “PET” OR “single photon emission computed tomography” OR “SPECT”). Lihua Gu and Hao Shu performed the literature search.

### Inclusion and exclusion criteria

The study included neuroimaging articles exploring brain changes of ICD in PD. In addition, included studies provided information for Talairach or Montreal Neurologic Institute (MNI) coordinates for comparisons between PD-ICD and PD-no ICD. Reviews, case reports and meta-analysis were excluded. Additionally, region of interest (ROI) analyses were excluded.

### Data collection

Lihua Gu and Hao Shu conducted the data collection. When discrepancies happened, data collection was discussed and decided by the three authors (Lihua Gu, Hao Shu and Yanjuan Wang). We extracted data from included studies: author and publication year, imaging modality, method, sample size, mean age, gender, unified Parkinson's disease rating scale (UPDRS)-score, disease duration (years), medication status, general cognitive status, ICD type, group contrasts and foci, correction for multiple comparisons and covariates.

### Meta-analysis

GingerALE Version 3.0.2 (http://www.brainmap.org/ale) was used to make ALE study. Talairach coordinates were converted to MNI coordinates using icbm2tal (Lancaster et al., 2007; Laird et al., 2010). Foci data were collected from included studies and recorded in a text file. Then, foci data were read into the GingerALE software. Thirdly, Permutation test (5000 permutations) on randomly distributed foci was conducted to acquire statistical significance. Full-width-half-maximum (FWHM) was calculated on the basis of sample size in every study (Turkeltaub et al., 2012). ALE maps were thresholded at *p* < 0.05 using the false discovery rate (FDR) with an extent threshold of 200 mm^3^. Lastly, ALE maps were overlaid onto the MNI 152 template and viewed ALE maps with Mango software (http://ric.uthscsa.edu/mango/mango.html).

## Results

### Search results

**Figure 1:**
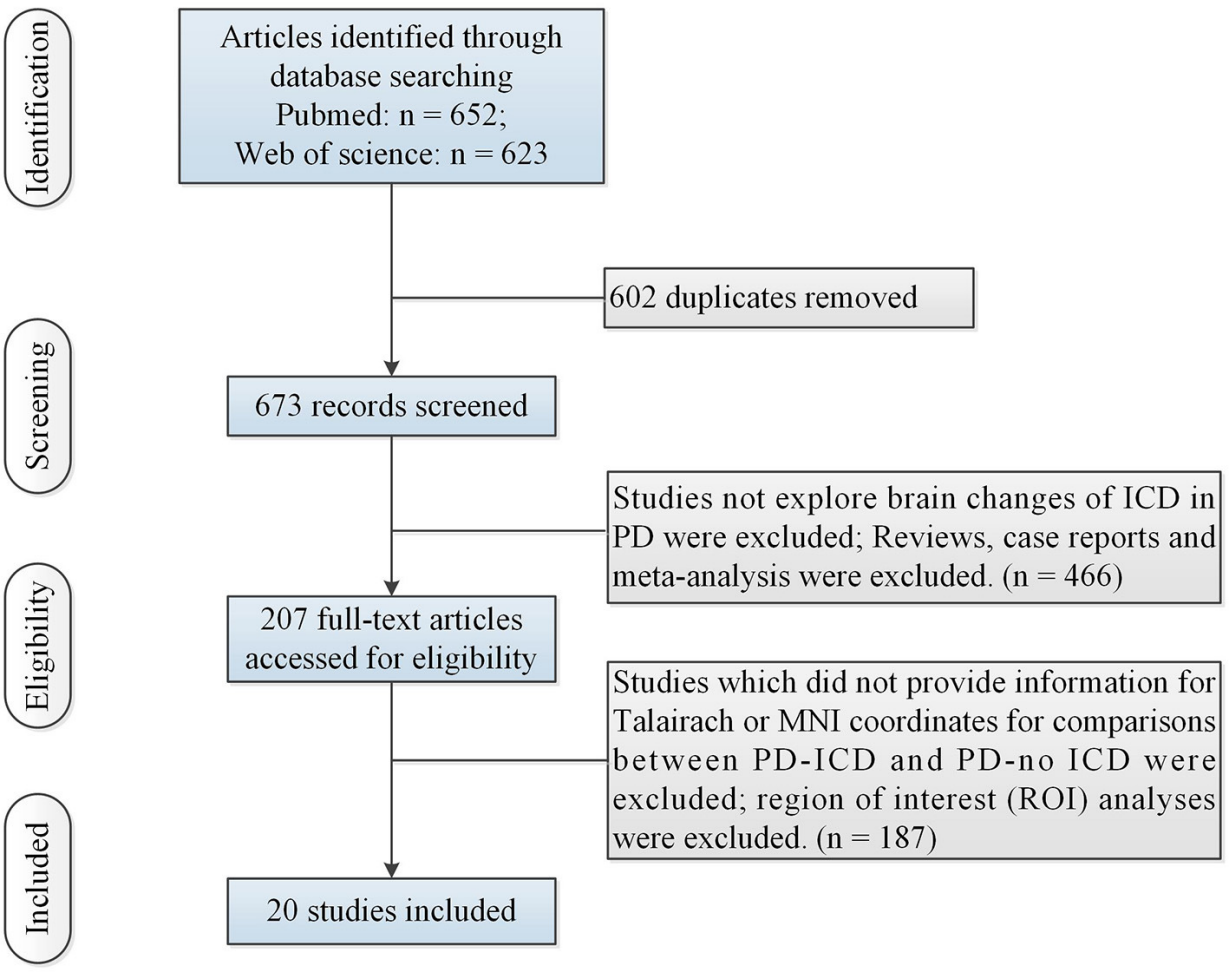
Flow of information through the different phases of the ALE study. ALE, activation likelihood estimation; ICD, impulse control disorder; MNI, Montreal Neurologic Institute; PD, Parkinson's disease.

Table 1 showed study characteristics. Figure 1 illustrated the initial search results and selection process. The study included 20 studies (Cilia et al., 2008; Frosini et al., 2010; Rao et al., 2010; van Eimeren et al., 2010; Voon et al., 2011; Ray et al., 2012; Cerasa et al., 2014; Lee et al., 2014; Biundo et al., 2015; Yoo et al., 2015; Premi et al., 2016; Tessitore et al., 2016, 2017a,b; Markovic et al., 2017; Filip et al., 2018; Imperiale et al., 2018; Verger et al., 2018; Esteban-Peñalba et al., 2021; Gan et al., 2021) (including 341 PD-ICD and 437 PD-no ICD).

**Table 1:** Characteristics of fMRI studies.

**Study**	**Imaging modality**	**Method**	**N (PD-ICDs/ PD-NO ICD)**	**Age (SD)**	**Gender (male%)**	**UPDRS-score**	**Disease duration (years)**	**Treatment**	**General** **Cognitive Status**	**ICD type**	**Group contrasts and Foci**	**Correction for multiple comparisons**	**Covariates**
**Structural MRI**													
Cerasa et al. (2014)	Structural MRI	VBM	12/12	58 ± 8.3	91.7%	UPDRSIII 26.4 (10.3)	NR	LEDD: 318.8 (150-800) mg	MMSE: 28.1 (1.5)	PG ICD diagnosed with G-SAS; BIS-11	PD-ICD < PD-NO ICD: 1	P ≤ 0.05 (FWE)	NR
Biundo et al. (2015)	Structural MRI	brain cortical thickness	58/52	60.3 (9.3)	65.5%	UPDRSIII 26.7 (16.5)	9.0 (5.5)	LEDD: 923.1 (474.1) mg; DA daily dosage:163.7 (111.3) mg	MMSE: 26.4 (2.6)	ICD diagnosed with DSM IV; MIDI; QUIP-RS	PD-ICD < PD-NO ICD: 14	P ≤ 0.05 (FDR)	Disease duration and LEDD
Tessitore et al. (2016)	Structural MRI	brain cortical thickness	15/15	62.87 (8.6)	NR	UPDRSIII 10.9 (4.5)	5.3 (2.9)	Total LEDD: 477.3 (222.9) mg; DA daily dosage: 243.3 (82.1) mg	MMSE: 26.5 (2.2)	HS; CE; PG; P ICD diagnosed with MIDI	PD-ICD < PD-NO ICD: 1; PD-ICD > PD-NO ICD: 5	P ≤ 0.01 (FDR)	LEDD and neuropsychological data differing between patients subgroups
Markovic et al. (2017)	Structural MRI	brain cortical thickness	22/30	63.1 (9.2)	86%	UPDRSIII 43.1±13.7	9.1±5.4	LEDD: 887.9±348.3 mg; DA daily dosage: 269.1 (141.2) mg	NR	P ICD diagnosed with PRS	PD-ICD < PD-NO ICD: 3;	P ≤ 0.05 (FDR)	age
**DTI**													
Yoo et al. (2015)	DTI	FA and MD	10/9	54.5 (6.2)	70%	UPDRSIII 14.6 (11.5)	10.2 (7.3)	LEDD: 924.6 (362.1) mg; DA: 255.0 (177.6)	MMSE: 28.0 (1.3)	ICD diagnosed with DSM IV	PD-ICD > PD-NO ICD: 1	P ≤ 0.05	NR
**fMRI**													
Frosini et al. (2010)	Task fMRI	BOLD response	7/7	57.5±11.1	NR	UPDRSIII 15.5±1.3	NR	LEDD: 462± 228.7 mg; DA: 408.3±156.3 mg	MMSE: 29.6	NR	PD-ICD > PD-NO ICD: 10	P ≤ 0.01 (FDR)	NR
Rao et al. (2010)	Rs-fMRI and Task fMRI	rCBF	9/9	56.2 (10.7)	77.8%	NR	7.2 (4.4)	LEDD: 418 (306) mg; DA: 278 (116) mg	MoCA: 27.1 (1.8)	pathological gambling, compul- sive sexual behavior and buying, binge-eating disorder,	Task fMRI: PD-ICD < PD-NO ICD: 1	P ≤ 0.05 (FDR)	NR
Voon et al. (2011)	Task fMRI	BOLD	14/ 14	51.52 (8.33)	71.4%	NR	NR	Total LEDD: 589.32 (301.25) mg; DA daily dosage: 161.53 (43.35) mg	MMSE: 27.73 (3.12)	PG; CS ICD diagnosed with DSM IV.	PD-ICD > PD-NO ICD: 3	P ≤ 0.05 (FDR)	NR
Tessitore et al. (2017a)	Rs-fMRI	FC	15/15	57 (9.7)	33.3%	UPDRSIII 15.7 (6)	1.4 (0.5)	LEDD: 202.7 (58.1) mg; DA daily dosage: 96 (65.5) mg	MMSE: 28.8 (8.6); MoCA: 24.6 (4.1)	HS; CE; PG; CS ICD diagnosed with QUIP-RS	PD-ICD < PD-NO ICD: 3; PD-ICD > PD-NO ICD: 1	NR	NR
Tessitore et al. (2017b)	Rs-fMRI	FC	15/15	62.87 (8.6)	86.6%	UPDRSIII 10.9 (4.5)	9.8 (5)	LEDD: 477.3 (222.9) mg; DA daily dosage: 243.3 (82.1) mg	MMSE: 26.5 (2.2)	HS; CE; PG; P ICD diagnosed with MIDI	PD-ICD < PD-NO ICD: 2; PD-ICD > PD-NO ICD: 4	P ≤ 0.05 (FWE)	NR
Filip et al. (2018)	Task fMRI	BOLD	8/13	65 (5.7)	75%	NR	9.75 (3.99)	LEDD: 1061.88 (270.7) mg	NR	ICD diagnosed with BIS	PD-ICD < PD-NO ICD: 2	P ≤ 0.05 (FWE)	NR
Imperiale et al. (2018)	Rs-fMRI	FC	35/ 50	62 (10.4)	85.7%	UPDRSIII 7.2 (15.5)	9.5 (5.2)	LEDD: 966.3 (438.7) mg; DA daily dosage: 228.14 (163.99) mg	MMSE: 28.3 (1.8); ACE-R: 90.0 (7.4)	HS; PG; CS; CE; P; DDS; ICD diagnosed with QUIP-RS	PD-ICD < PD-NO ICD: 5	P ≤ 0.05 (FWE)	age
Esteban-Peñalba et al. (2021)	Task fMRI	BOLD	18/17	63.33 (8.24)	88.90%	UPDRSIII 21.50 [10–46]	7.13 (3.96)	LEDD:970 mg; DA: 194.83 (165.99) mg	NR	ICD diagnosed with DSM IV	PD-ICD < PD-NO ICD: 2; PD-ICD > PD-NO ICD: 2	P ≤ 0.05 (FWE)	NR
Gan et al. (2021)	Rs-fMRI	VMHC	21/33	59.0±9.6	57.1%	UPDRSIII 20.3±14.2	9.0±5.2	LEDD: 770.7±310.8mg; DA: 90.5±57.4 mg	MMSE: 28.3±1.1	PG, HS, BE, and CS ICD diagnosed with DSM IV	PD-ICD < PD-NO ICD: 1	P < 0.01	age, sex, degree of education
**PET**													
van Eimeren et al. (2010)	PET	rCBF	7/7	60 (9.8)	100%	UPDRS off: 28.3 (7.5); UPDRS on: 20.1 (3.7)	7.3 (3)	Total LEDD: 771.7 (318.4) mg; DA daily dosage: 143.4 (105.2) mg	MoCA: 27.1 (2.5)	PG ICD diagnosed with G-SAS	PD-ICD < PD-NO ICD: 5	NR	NR
Ray et al. (2012)	PET	rCBF	7/7	59.71 (10.98)	100%	UPDRSIII 21 (8.04)	NR	Total LEDD: 888.29 (479.96) mg	NR	PG ICD diagnosed with G-SAS; BIS	PD-ICD > PD-NO ICD: 1	P ≤ 0.05 (FWE)	NR
Lee et al. (2014)	PET	rCBF	11/11	56.6 (8.7)	72.7%	UPDRSIII 14.2 (11)	10.1 (6.9)	Total LEDD: 914.4 (338.7) mg; DA daily dosage: 217.9 (175.3) mg	MMSE: 27.7 (1.6)	HS; PG; CE; CS; P; ICD diagnosed with DSM IVTR; MIDI	PD-ICD < PD-NO ICD: 2; PD-ICD > PD-NO ICD: 4	P < 0.005	NR
Verger et al. (2018)	PET	rCBF	18/18	60.4 (67.3)	83.3%	UPDRS off: 31.9 (12.0); UPDRS on: 5.3 (4.1)	10.96 (3.6)	LEDD: 1124.1 (320.5) mg; DA daily dosage: 157.3 (130.0) mg	NR	PG; CS; HS ICD diagnosed with DSM IVTR; MIDI	PD-ICD < PD-NO ICD: 2; PD-ICD > PD-NO ICD: 3	P < 0.005	NR
**SPECT**
Cilia et al. (2008)	SPECT	rCBF	11/40	57.4 (5.8)	90.9%	UPDRSIII 18 (11)	8.4 (3.4)	Total LEDD: 811.8 (229.0) mg; DA daily dosage: 289.1 (57.5) mg	MMSE: 28.9 (0.8)	PG ICD diagnosed with DSM IVTR	PD-ICD < PD-NO ICD: 7	P ≤ 0.05 (FDR)	NR
Premi et al. (2016)	SPECT	rCBF	21/63	65.8 (8.4)	23.8%	UPDRSIII 16.5 (7.2)	1.9 (2.2)	Total LEDD: 594.2 (388.6) mg; DA daily dosage: 282.1 (227.9) mg	MMSE: 27.9 (1.9)	CE; PG; HS; P ICD diagnosed with QUIP-RS	PD-ICD < PD-NO ICD: 2	P < 0.005	age, gender, disease duration and clinical phenotype

Abbreviations: BIS, Barratt Impulsivity Scale; BOLD, blood oxygen level dependent; CE, compulsive eating; CS, compulsive buying; DA, dopamine agonists; DTI, diffusion tensor image; DSM, diagnostic and statistical manual of mental disorders; FA, fractional anisotropy; FC, functional connectivity; FDR, false discovery rate; FWE, family-wise error; G-SAS, Gambling Symptom Assessment Scale; HS, hypersexuality; ICD, impulse control disorder; LEDD, levodopa equivalent daily dose; MD, mean diffusivity; MIDI, Minnesota Impulsive Disorders Interview; MMSE, Mini-Mental State Examination; MoCA, Montreal Cognitive Assessment; N, sample size; P, punding; PD, Parkinson's disease; PET, positron emission tomography; PG, pathological gambling; PRS, Punding Rating Scale; QUIP-RS, questionnaire for impulsive-compulsive disorders in Parkinson's disease rating scale; rCBF, regional cerebral blood flow; Rs-fMRI, resting-state functional magnetic resonance imaging; SPECT, single photon emission computed tomography; UPDRS, Unified Parkinson's Disease Rating Scale; VBM, voxel-based morphometry.

### Results of meta-analysis

#### Abnormal gray and white matter in PD-ICD

**Figure 2:**
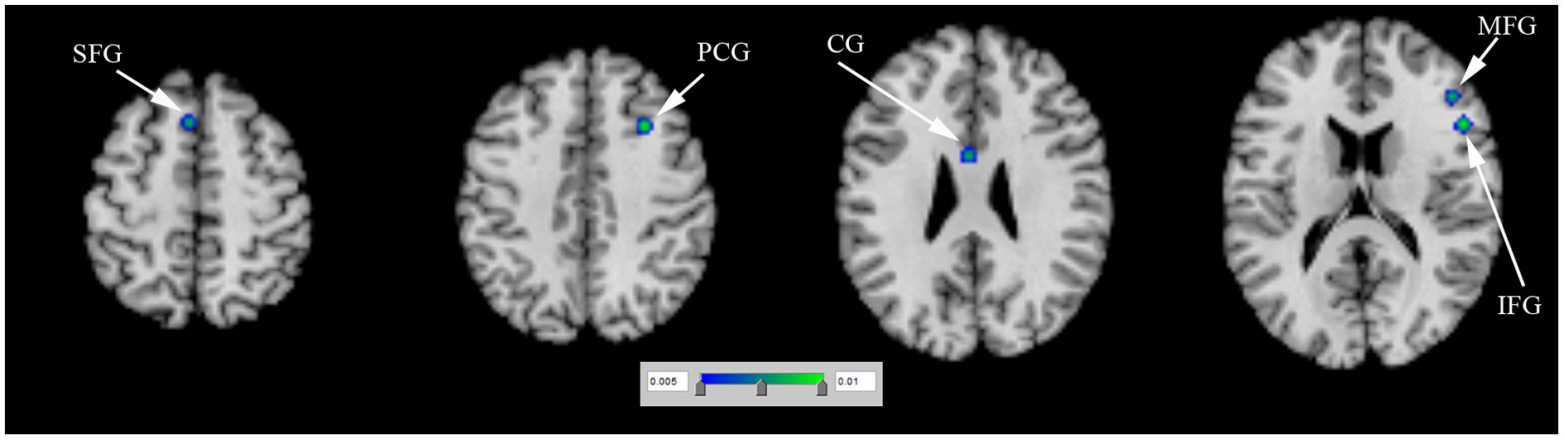
Thinner cortical thickness in PD-ICD patients, compared to PD-no ICD (in blue). CG, cingulate gyrus; ICD, impulse control disorder; IFG, inferior frontal gyrus; MFG, middle frontal gyrus; PCG, precentral gyrus; PD, Parkinson's disease; SFG, superior frontal gyrus.

*N* = 1 study (Cerasa et al., 2014) (including 12 PD-ICD and 12 PD-no ICD patients) made whole-brain voxel-based morphometry (VBM) analysis of PD-ICD related gray matter (GM) abnormalities. VBM study showed gray matter volume loss in the orbitofrontal cortex (OFC) in PD-ICD, compared to PD-no ICD patients. *N* = 3 studies (Biundo et al., 2015; Tessitore et al., 2016; Markovic et al., 2017) (including 95 PD-ICD and 97 PD-no ICD patients) explored changes of brain cortical thickness in PD-ICD. PD-ICD patients showed significant cortical thinning in the right IFG, the right middle frontal gyrus (MFG), the left superior frontal gyrus (SFG), the right precentral gyrus (PCG) and the left cingulate gyrus (CG) (Figure 2 and Supplementary table S2), compared to PD-no ICD patients. *N* = 1 study (Yoo et al., 2015) (including 10 PD-ICD and 9 PD-no ICD patients) indicated significantly higher fractional anisotropy (FA) in the anterior corpus callosum, right internal capsule posterior limbs, right posterior cingulum, and right thalamic radiations in PD-ICD patients, compared to PD-no ICD patients, whereas no significant difference in mean diffusivity (MD) was showed in any brain regions between the two groups.

#### Abnormal resting-state activation in PD-ICD

**Figure 3:**
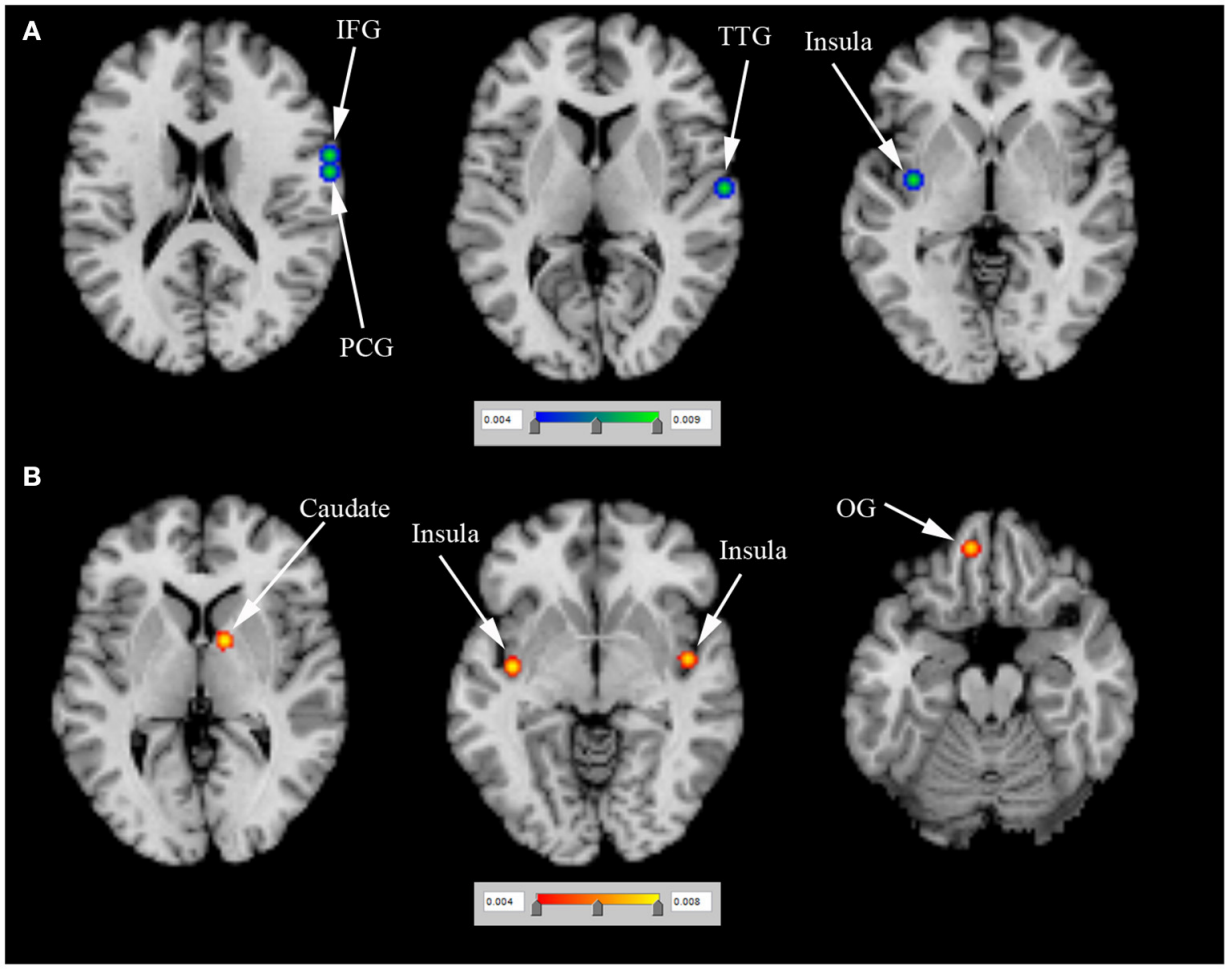
**(A)** Reduced resting-state brain activation (in blue) and **(B)** increased resting-state brain activation (in red) in PD-ICD patients, compared to PD-no ICD. ICD, impulse control disorder; IFG, inferior frontal gyrus; OG, orbital gyrus; PCG, precentral gyrus; PD, Parkinson's disease; TTG, transverse temporal gyrus.

*N* = 1 study (Rao et al., 2010) (including 9 PD-ICD and 9 PD-no ICD patients) showed significantly reduced regional cerebral blood flow (rCBF) in the right ventral striatum in PD-ICD, compared to PD-no ICD patients. *N* = 4 studies (Tessitore et al., 2017a,b; Imperiale et al., 2018; Gan et al., 2021) (including 86 PD-ICD and 113 PD-no ICD patients) compared resting-state brain activation between PD-ICD and PD-no ICD patients. The ALE study showed reduced resting-state brain activation in the right IFG, the right PCG, the left insula and the right transverse temporal gyrus (TTG) in PD-ICD, compared to PD-no ICD patients (Figure 3A and Supplementary table S2). PD-ICD showed increased resting-state brain activation in the right caudate, (Figure 3B and Supplementary table S2).

#### Abnormal task-related activation in PD-ICD

**Figure 4:**
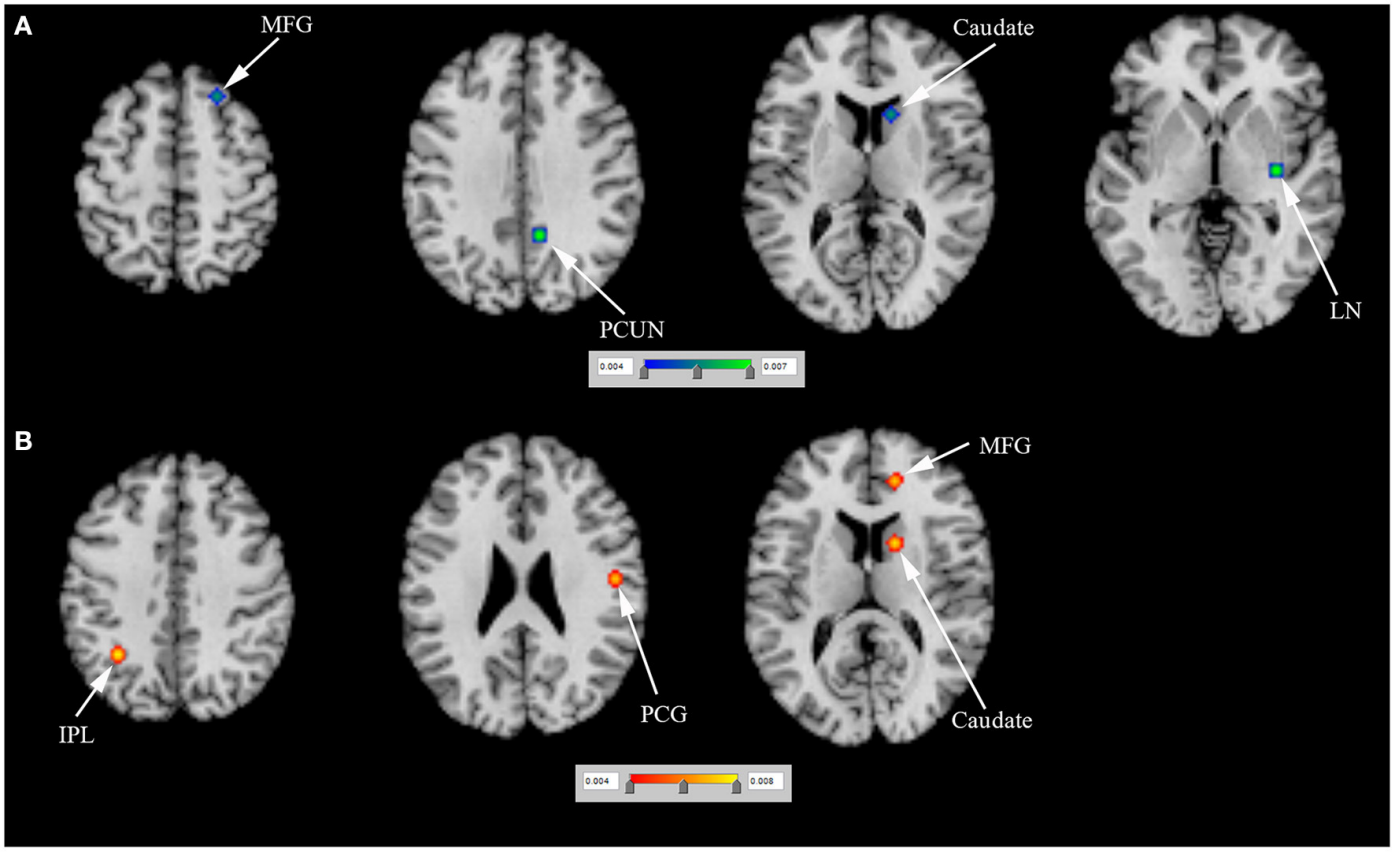
**(A)** Reduced task-related brain activation (in blue) and **(B)** increased task-related brain activation (in red) in PD-ICD patients, compared to PD-no ICD. ICD, impulse control disorder; IPL, inferior parietal lobule; LN, lentiform nucleus; MFG, middle frontal gyrus; PCG, precentral gyrus; PCUN, precuneus; PD, Parkinson's disease.

*N* = 5 studies (Frosini et al., 2010; Rao et al., 2010; Voon et al., 2011; Filip et al., 2018; Esteban-Peñalba et al., 2021) (including 56 PD-ICD and 60 PD-no ICD patients) compared task-related brain activation between PD-ICD and PD-no ICD patients. The ALE study indicated reduced task-related brain activation in the right caudate, the right MFG, the right lentiform nucleus (LN) and the right PCUN in PD-ICD, compared to PD-no ICD patients (Figure 4A and Supplementary table S2). The ALE study showed increased task-related brain activation in the left inferior parietal lobule (IPL), the right medial frontal gyrus, the right caudate and the right PCG in PD-ICD, compared to PD-no ICD patients (Figure 4B and Supplementary table 2).

#### Abnormal rCBF in PD-ICD

**Figure 5:**
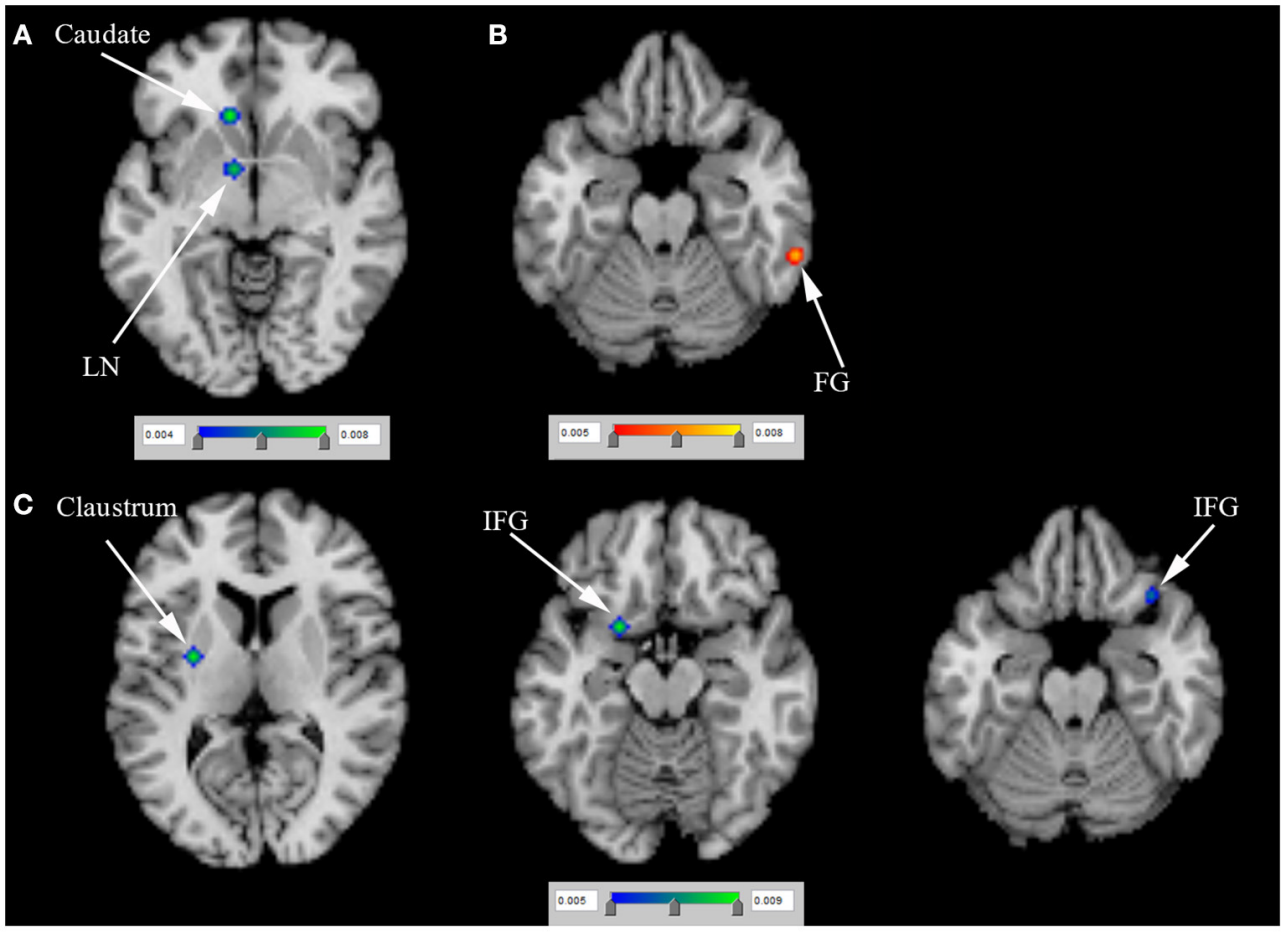
**(A)** Reduced rCBF (in blue), **(B)** increased rCBF (in red) using PET and **(C)** reduced rCBF (in blue) using SPECT in PD-ICD patients, compared to PD-no ICD. FG, fusiform gyrus; ICD, impulse control disorder; IFG, inferior frontal gyrus; LN, lentiform nucleus; PD, Parkinson's disease; PET, positron emission tomography; rCBF, regional cerebral blood flow; SPECT, single photon emission computed tomography.

*N* = 4 positron emission tomography (PET) studies (van Eimeren et al., 2010; Ray et al., 2012; Lee et al., 2014; Verger et al., 2018) (including 43 PD-ICD and 43 PD-no ICD patients) compared rCBF between PD-ICD and PD-no ICD patients. The ALE study showed reduced rCBF in the left LN and the left caudate in PD-ICD, compared to PD-no ICD patients using PET (Figure 5A and Supplementary table S2). In addition, the ALE study showed increased rCBF in the right fusiform gyrus (FG) in PD-ICD, compared to PD-no ICD patients using PET (Figure 5B and Supplementary table S2). *N* = 2 single photon emission computed tomography (SPECT) studies (Cilia et al., 2008; Premi et al., 2016) (including 39 PD-ICD and 103 PD-no ICD patients) compared rCBF between PD-ICD and PD-no ICD patients. The ALE study indicated that PD-ICD showed reduced rCBF in the bilateral IFG and the left claustrum using SPECT (Figure 5C and Supplementary table S2).

## Discussion

### Abnormal cortical thickness in PD-ICD

Frontostriatal circuit plays a prominent role in ICD (Pellicano et al., 2015). The present ALE study showed that cortical thinning of the right IFG was of most particular interest in the comparison of cortical thickness between PD-ICD and PD-no ICD. Right IFG is a specifically functional brake of an unuseful behavior (Aron et al., 2014). MFG is related to rule-related behavior, working memory and making strategic decisions (Simmonds et al., 2008; Steinbeis et al., 2012). SFG inhibited behavioral bias between immediate rewards and long-term goals (Diekhof and Gruber, 2010). PCG and CG play a key role in the executive control of cognition (Carter et al., 1998; Liu et al., 2021). Brain changes in IFG, MFG, SFG, PCG and CG contribute to ICD in PD.

### Abnormal resting-state and task-related activation in PD-ICD

Previous study demonstrated that the insula works as a key node in detection and selection of salient events (Singer et al., 2009). Insula shows additional processing to create an effective behavioral response (Singer et al., 2009). TTG, together with insula, plays a critical role in the emotional memory and increases the reward sensitivity (Moreno-Lopez et al., 2016). The caudate is associated with response inhibition (Hardee et al., 2014). Caudate is one of reward-related regions responding to general reward processing (Wang et al., 2021). OG worked as a key node in motor inhibition network, which could inhibit the response to incongruous stimulation (Hardee et al., 2014). LN moderates reward and value-based decision making (Sperling and Müller, 2011). Moreover, PCUN and IFL are needed to initiate preferential allocation of attentional resources in preparation for actions elicited by craving (Folstein et al., 1975). The present study supported brain changes in the IFG, PCG, insula, TTG, caudate, OG, MFG, LN, PCUN, IPL and PCG in PD-ICD.

### Abnormal rCBF in PD-ICD

Until now, the role of the FG is still not clear. Previous studies supported that the activity of FG may be essential for encoding visual objects (George et al., 1999) and subsequent memory (Haxby et al., 2001). The activity of FG increases the reward sensitivity (Haxby et al., 2001). Terem et al. (2020) reported that the claustrum worked as a critical part of the reward system, especially in an incentive salience process involved in cue-reward association. The present study showed brain changes in the LN, caudate, FG, IFG and claustrum in PD-ICD.

The present study showed some limitations. Firstly, ALE study could not investigate heterogeneities between included studies. Secondly, ALE study could not assess the significance level of contributing results. Thirdly, publication bias between included studies could not be explored. Fourthly, only *N* = 20 studies were included. The small sample sizes may have reduced the power of the analysis.

## Conclusions

The present ALE analysis has confirmed that brain changes in frontal, temporal and basal ganglia regions are among the most frequently reported regions in PD-ICD. Deficits in these regions could play a role in diagnosis of PD-ICD.

## Data availability statement

The original contributions presented in the study are included in the article/Supplementary material, further inquiries can be directed to the corresponding author/s.

## Author contributions

LG: manuscript writing, study search, data collection, software use, data analysis, funding support, and manuscript revision. HS: study search, data collection, and manuscript revision. YW: data collection, software use, and funding support. HX: data analysis. All authors contributed to the article and approved the submitted version.

## Funding

This study was supported by the National Natural Science Foundation of China (No. 81901108).

## Conflict of interest

The authors declare that the research was conducted in the absence of any commercial or financial relationships that could be construed as a potential conflict of interest.

## Publisher's note

All claims expressed in this article are solely those of the authors and do not necessarily represent those of their affiliated organizations, or those of the publisher, the editors and the reviewers. Any product that may be evaluated in this article, or claim that may be made by its manufacturer, is not guaranteed or endorsed by the publisher.
