# Supplementary material for: Exploring brain changes of impulse control disorders in Parkinson's disease: An ALE study
Source: Front Aging Neurosci. 2022 Aug 30;14:966525. doi: 10.3389/fnagi.2022.966525 (PMC9468821; doi:10.3389/fnagi.2022.966525)
Supplement: Supplementary file 2 [file Table_2.docx]

Supplementary table 2. ALE results for neuroimaging studies.

| Cluster # | Volume (mm^3^) | Weighted Center (MNI) | | | Extrema value | Maximum ALE value (MNI) | | | Side | BA | Anatomical label |
| --- | --- | --- | --- | --- | --- | --- | --- | --- | --- | --- | --- |
|  |  | x | y | z |  | x | y | z |  |  |  |
| **ALE results for structural MRI regarding brain cortical thickness (95 PD-ICD and 97 PD-no ICD patients)** | | | | | | | | | | | |
| PD-ICD < PD-no ICD (17 foci, 3 experiments) | | | | | | | | | | | |
| 1 | 704 | 53.3 | 21.9 | 15.8 | 0.00999937 | 50 | 20 | 18 | Right | 9 | Inferior Frontal Gyrus |
| 2 | 384 | 36.5 | 10.1 | 51 | 0.009687987 | 36 | 10 | 50 | Right | 5 | Middle Frontal Gyrus |
| 3 | 376 | -4.9 | 20.9 | 54 | 0.0096 | -4 | 20 | 54 | Left | 6 | Superior Frontal Gyrus |
| 4 | 368 | 50.2 | 3.2 | 35.5 | 0.0098 | 50 | 4 | 36 | Right | 6 | Precentral Gyrus |
| 5 | 320 | -2.9 | 4.6 | 27.4 | 0.0083 | -2 | 4 | 28 | Left | 24 | Cingulate Gyrus |
| **ALE results for resting-state fMRI studies (86 PD-ICD and 113 PD-no ICD patients)** | | | | | | | | | | | |
| PD-ICD < PD-no ICD (12 foci, 4 experiments) | | | | | | | | | | | |
| 1 | 896 | 61.9 | 2.1 | 20 | 0.0095 | 62 | 6 | 20 | Right | 44 | Inferior Frontal Gyrus |
| 2 | 768 | 62 | -2 | 20 | 0.0096 | 62 | -2 | 20 | Right | 6 | Precentral Gyrus |
| 3 | 456 | -38 | -6 | 0 | 0.0094 | -38 | -6 | 0 | Left | 13 | Insula |
| 4 | 456 | 62 | -10 | 8 | 0.0094 | 62 | -10 | 8 | Right | 42 | Transverse Temporal Gyrus |
| PD-ICD > PD-no ICD (5 foci, 2 experiments) | | | | | | | | | | | |
| 1 | 424 | 10.9 | 4.5 | 3.8 | 0.0075 | 10 | 4 | 4 | Right |  | Caudate |
| 2 | 416 | -41 | -8.4 | -4.2 | 0.0075 | -42 | -8 | -4 | Left | 13 | Insula |
| 3 | 408 | 44.3 | -4.6 | -2.8 | 0.0075 | 44 | -4 | -2 | Right | 13 | Insula |
| 4 | 320 | -10 | 49.1 | -18.6 | 0.0076 | -10 | 50 | -20 | Left | 11 | Orbital Gyrus |
| **ALE results for task-related fMRI studies (56 PD-ICD and 60 PD-no ICD patients)** | | | | | | | | | | | |
| PD-ICD < PD-no ICD (4 foci, 2 experiments) | | | | | | | | | | | |
| 1 | 456 | 12 | 18 | 12 | 0.006 | 12 | 18 | 12 | Right |  | Caudate |
| 2 | 456 | 22 | 28 | 56 | 0.006 | 22 | 28 | 56 | Right | 6 | Middle Frontal Gyrus |
| 3 | 448 | 33 | -13 | 1 | 0.0074 | 33 | -13 | \| 1 \| 1 \| \| --- \| --- \| | Right |  | Lentiform Nucleus |
| 4 | 448 | 9 | -49 | 37 | 0.0074 | 9 | -49 | 37 | Right | 31 | Precuneus |
| PD-ICD > PD-no ICD (15 foci, 3 experiments) | | | | | | | | | | | |
| 1 | 480 | 51 | -10 | 28 | 0.0079 | 51 | -10 | 28 | Right | 6 | Precentral Gyrus |
| 2 | 448 | -33 | -52 | 43 | 0.0077 | -32 | -52 | 44 | Left | 40 | Inferior Parietal Lobule |
| 3 | 416 | 14.2 | 44.4 | 15.3 | 0.0075 | 14 | 44 | 16 | Right | 9 | Medial Frontal Gyrus |
| 4 | 392 | 14.4 | 9.6 | 14.5 | 0.0075 | 14 | 10 | 14 | Right |  | Caudate |
| **ALE results for PET studies (43 PD-ICD and 43 PD-no ICD patients)** | | | | | | | | | | | |
| PD-ICD < PD-no ICD (9 foci, 3 experiments) | | | | | | | | | | | |
| \| 1 \| 680 \| -13 \| 2 \| -8 \| 0.0071 \| -10 \| 2 \| -4 \| Left \|  \| Lentiform Nucleus \| \| --- \| --- \| --- \| --- \| --- \| --- \| --- \| --- \| --- \| --- \| --- \| --- \| \| 2 \| 400 \| -12 \| 27.1 \| -4.9 \| 0.0078 \| -12 \| 28 \| -4 \| Left \|  \| Caudate \| | | | | | | | | | | | |
| PD-ICD > PD-no ICD (9 foci, 3 experiments) | | | | | | | | | | | |
| \| 1 \| 680 \| 64.3 \| -38.7 \| -23.2 \| 0.008 \| 62 \| -38 \| -22 \| Right \| 20 \| Fusiform Gyrus \| \| --- \| --- \| --- \| --- \| --- \| --- \| --- \| --- \| --- \| --- \| --- \| --- \| | | | | | | | | | | | |
| **ALE results for SPECT studies (39 PD-ICD and 103 PD-no ICD patients)** | | | | | | | | | | | |
| PD-ICD < PD-no ICD (9 foci, 2 experiments) | | | | | | | | | | | |
| \| 1 \| 264 \| -22 \| 10 \| -14 \| 0.0085 \| -22 \| 10 \| -14 \| Left \| 47 \| Inferior Frontal Gyrus \| \| --- \| --- \| --- \| --- \| --- \| --- \| --- \| --- \| --- \| --- \| --- \| --- \| \| 2 \| 264 \| -30 \| -4 \| 10 \| 0.0085 \| -30 \| -4 \| 10 \| Left \|  \| Claustrum \| \| 3 \| 200 \| 35.6 \| 24.7 \| -21.1 \| 0.0065 \| 36 \| 24 \| -22 \| Right \| 47 \| Inferior Frontal Gyrus \| | | | | | | | | | | | |

Abbreviations: ALE, anatomic likelihood estimation; BA, Brodmann area; fMRI, functional magnetic resonance imaging; ICD, impulsive control disorders; MNI, montreal neurologic institute; PD, Parkinson’s disease; PET, positron emission tomography; SPECT, .single photon emission computed tomography.
